# Virtual reality modulates the control of upper limb motion in one-handed ball catching

**DOI:** 10.3389/fspor.2022.926542

**Published:** 2022-10-06

**Authors:** Hirofumi Ida, Kazunobu Fukuhara, Takahiro Ogata

**Affiliations:** ^1^Department of Sports and Health Management, Jobu University, Isesaki, Japan; ^2^Department of Health Promotion Science, Tokyo Metropolitan University, Hachioji, Japan; ^3^Department of Sport and Medical Science, Teikyo University, Hachioji, Japan

**Keywords:** virtual reality, physical reality, CAVE, interceptive action, reaction time, electromyography, sense of presence, perception-action coupling

## Abstract

There remains a question about whether and to what extent perception–action coupled response in virtual reality are equal/unequal to those in the real world or physical reality. The purpose of this study was to identify the differences in the environmental effect of virtual presentation on the motor responses of a one-handed ball catching. Thirteen healthy participants were instructed to catch an approaching ball projected at three speeds in a real laboratory room and in a room-sized virtual reality system (CAVE) that simulated those real situations with two- or three-dimensional display settings. The results showed that the arm movement time, which denotes the duration of arm-raising motion (shoulder flexion), was significantly longer in the virtual reality than that in the physical reality at the fast ball speed condition. The shoulder flexion velocities, calculated as the average angular velocity of shoulder flexion over the arm movement time, were significantly lower in the virtual reality than in the physical reality at the medium and fast ball speed conditions. The electromyography onsets, derived from anterior deltoid, biceps brachii, and flexor carpi radialis muscles of the catching arm, appeared before and significantly closer to the initiation of arm raising in the two-dimensional virtual reality than both in the physical reality and in the three-dimensional virtual reality. The findings suggest that simulation of virtual reality may induce a modulation in the motor responses of the catching arm, which is different from natural motion that appeared in the real world. On the contrary, the effect of ball speed generally found in real setting was maintained in the current CAVE experiment.

## Introduction

Over the last few decades, virtual reality (VR) has become widely used in neuroscientific studies on human perceptual-motor control (1, 2). To effectively apply VR technologies to assess or improve motor skill, it is necessary to take into account whether and to what extent the neuromuscular control and resultant performance generated in the virtual setting are equal/unequal to those in the real world or physical reality (PR). A cave automatic virtual environment (CAVE) is a room-sized cubic VR system that is typically constructed with multiple screen walls and two projectors per wall for left and right eyes (3, 4). Unlike VR headset devices known as head-mounted display (HMD), users put on lightweight eyewear such as polarized glasses to view stereoscopic image and experience whole-body immersion in CAVE. When studying perceptual-motor activities like ball sports by utilizing VR environment, CAVE is one of the promising options (5). In general, human visual perceptual processing, for instance egocentric collision perception, is likely to be altered depending on the display setting of VR (6). Thus, it is reasonable to suppose that the motor responses are modulated in accordance with the quality of the virtual scene displayed (2).

Catching a ball is one of the visually guided interceptive actions that require close perception–action coupling with fine spatiotemporal control of end effector (7). Several previous studies conducted in real setting by using ball-projection machine clearly demonstrated the effect of ball speed on catching performance (8–10). To be specific, the number of successful trials decreased and the motor response time shortened with increasing ball speed. In early studies that assessed the performance of ball catching by utilizing VR, the main concern was to identify the mathematical model of catcher's running path, known as the “outfielder problem” (11, 12). In this context, it is concluded that the judgment and interception of fly ball can be successfully performed in CAVE in a similar manner with PR environment (13). Another study, however, clearly pointed out the dissimilarities between PR and VR settings in the kinematics of manual catching task by using a swinging ball suspended from the ceiling (14): the movement of catching hand in CAVE was initiated later, less accurate, smoother, and aimed more directly to the intercepting point as compared with that in an equivalent PR setting.

In addition to this, the dimensionality of visual presentation, or two-dimensional (2D) and three-dimensional (3D) display, possibly affects the visuo-motor coordination while catching an approaching ball (15, 16). It was shown that unnatural binocular viewing through a telestereoscope, by which effective interocular separation was increased, reduced the number of successful trials in one-handed catching task as compared with unimpaired binocular viewing (17). The subsequent studies further demonstrated that binocular information would be used for timing the hand closure movement (18) in combination with monocular information (19). On the other hand, it was also demonstrated that people with weak stereopsis showed less percentage of correct catches (9) and less improvement by catching training (10) as compared to those with normal stereopsis. These findings imply that the binocular setting of VR display, 2D or 3D, possibly has a critical impact on the catching performance. However, there are quite few studies that have addressed the effect of the dimensionality of VR display on the motor control of one-handed catching.

As mentioned above, kinematic analyses performed in a previous study successfully demonstrated that there were substantial discrepancies between PR and VR in the hand movement during catching (14). However, little is known about how such environmental effect emerges as muscle responses in catching arm. For example, analysis of reaction time based on the measurement of electromyography (EMG) onset is one of the simple but effective techniques to address these issues. In general, reaction time is fractionated into two components, that is, premotor time and motor time (20). The premotor time is measured as the temporal duration from the stimulus onset to the appearance of muscle action potential. On the contrary, the motor time is the duration from the firing of muscle action potential to the initiation of body segment/joint movement.

The purpose of this study was to demonstrate the environmental effect of VR on the motor responses of one-handed ball catching in comparison with that of PR. Furthermore, the effects of 2D or 3D displays configured in CAVE were also examined. To these ends, a natural and pseudo-natural catching task was adopted, where a projected real or virtual ball approached in depth, in a parabolic path, and in three speeds (8–10, 17). The assessment of motor response was performed by spatiotemporal analyses of the raising motion (shoulder flexion) of catching arm and temporal analyses of its EMG onsets. The primary hypothesis was that the raising motion and the EMG onset were different between the PR and VR settings. More specifically, it was expected that the movement time of arm raising was shortened in VR as inferred from a previous study (14). Secondarily, it was also hypothesized that the display setting of 2D or 3D could affect these motor responses. In this regard, the catching performance may be deteriorated by the 2D display as compared with the 3D display due to the lack of binocular depth information (9). A supplementary hypothesis was also formulated that the effect of ball speed was maintained in VR setting as in PR, which means that catching performance is deteriorated and reaction time is shortened with increasing ball speed.

## Materials and methods

### Participants

Thirteen male healthy volunteers (mean age = 20.2 years, SD = 1.4) participated in the study. The dominant hand of all participants was right (21). They had normal or corrected-to-normal vision and normal stereoacuity (Stereo Fly SO-001, Stereo Optical Company Inc., USA). The study was reviewed and approved by the institutional review boards of the Jobu University and Kanagawa Institute of Technology. All the participants provided their written informed consent to participate in this study.

### Apparatus and setting

#### Physical reality experiment

**Figure 1:**
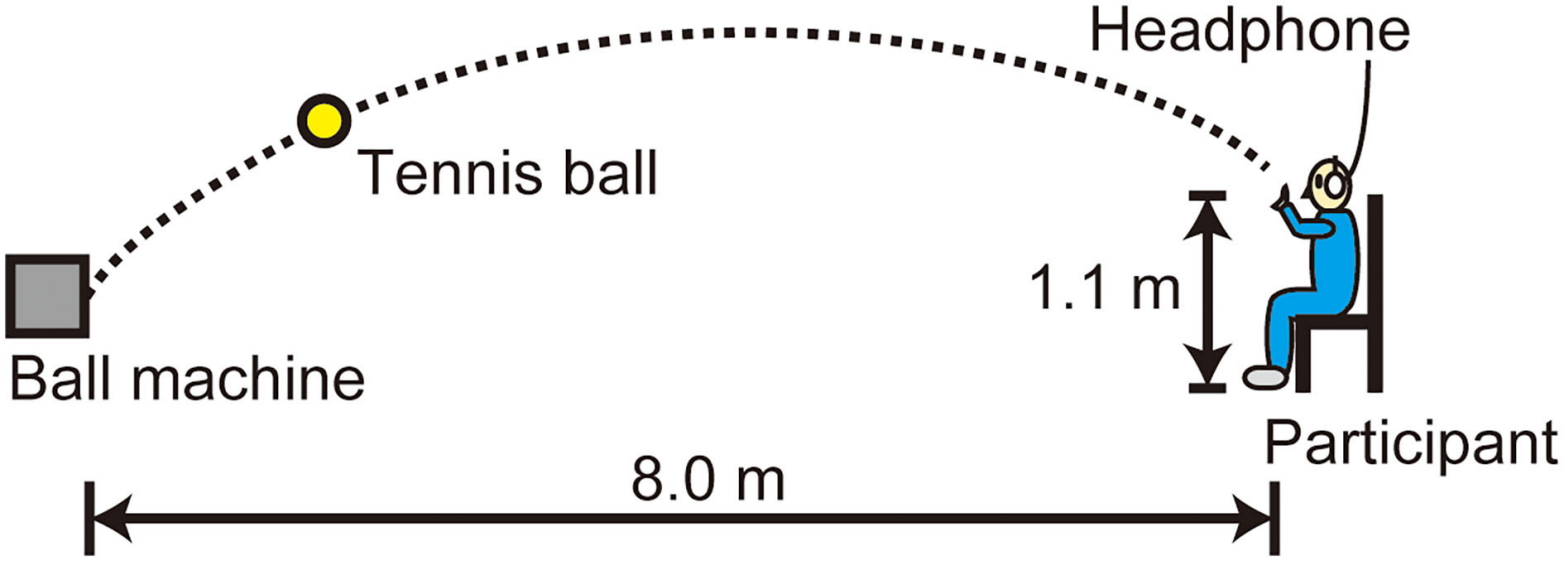
Setup of the PR experiment. The participants caught a ball projected from an automatic feeding machine with their non-dominant (left) hand.

The PR experiment used an automatic feeding machine (TENNIS TUTOR, Sports Tutor Inc., USA) to project tennis balls. The target of the ball was set at a distance of 8.0 m apart from the ball machine and a height of 1.1 m (Figure 1). The ball speed was set at slow, medium, and fast by turning the dial on the control panel: A preliminary measurement of 10 projections showed a mean velocity of 8.7 m/s (SD = 0.1), 11.7 m/s (SD = 0.3), and 15.3 m/s (SD = 0.3) on a radar gun (SPEEDSTER V, Bushnell Inc., USA), respectively. The ball was automatically launched at an interval of ~5 s, where the delays of ball delivery occurred irregularly. The participants put on a headphone (QuietComfort^®^3, Bose Inc., USA) that shut out the ambient sound.

A set of flashlight and photodetector was located at the ejection window of the ball machine to measure the moment of ball launch. An accelerometer (MA3, MicroStone Inc., Japan) was attached to the dorsum of the participants' catching hand to estimate the moment of ball-hand contact. The shoulder flexion angle was measured using a goniometer (SG110, Biometrics Inc., UK). The surface EMG signals of anterior deltoid (AD), biceps brachii (BB), and flexor carpi radialis (FCR) muscles of the catching arm were collected using active probe electrodes (SX230W, Biometrics Inc., UK), by which the signal was band-pass-filtered (20 Hz−460 Hz) and amplified (gain 1,000). The photodetector, accelerometer, goniometer, and EMG signals were recorded using LabVIEW SignalExpress 2.0.0 software at a sampling rate of 1,000 Hz with a 16-bit digital resolution via an analog-to-digital converter (NI 9215 attached to cDAQ-9172, National Instruments Inc., USA).

#### Virtual reality experiment

**Figure 2:**
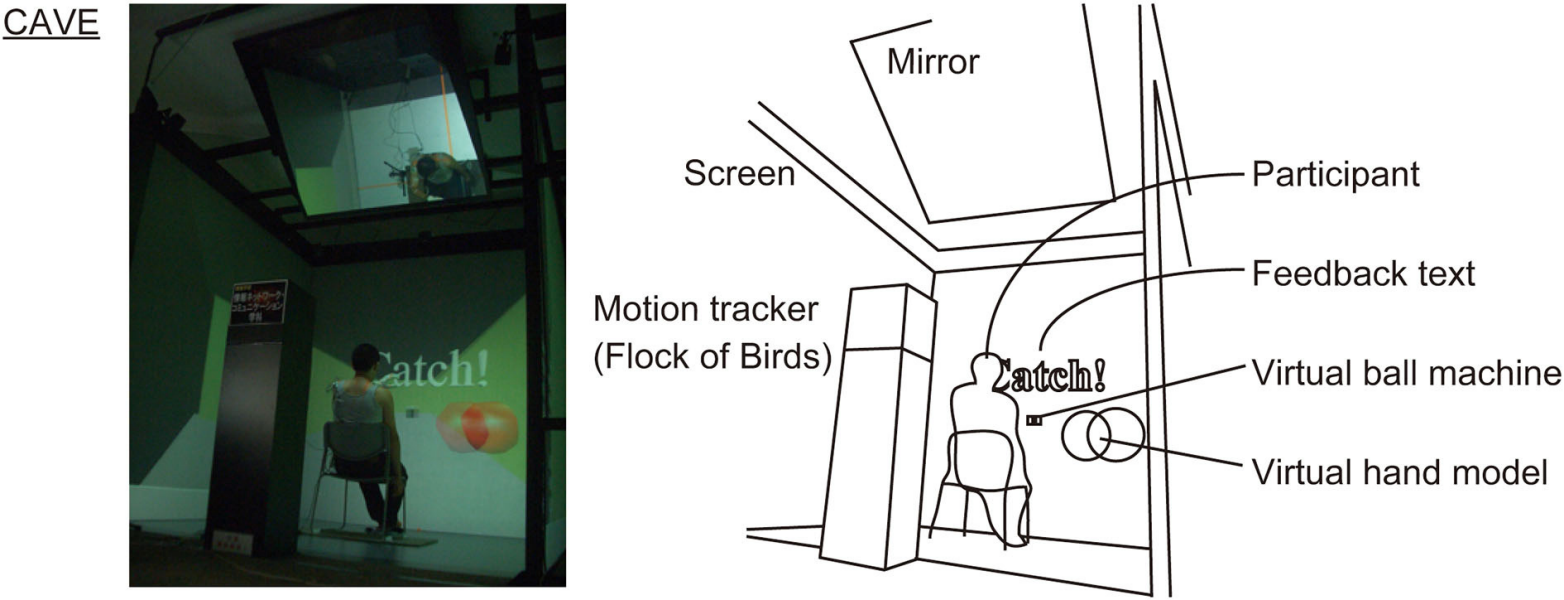
The VR experiment using CAVE. The virtual hand model and feedback text “Catch!” were invisible during trial. The feedback text was temporarily displayed after successful trials.

The VR experiment used a 3-wall-1-floor type CAVE (2.5 m × 2.5 m, 1,050 × 1,050 pixels for each plane; Solidray Inc., Japan; Figure 2). This CAVE was controlled by one main personal computer (PC) and eight sub-PCs (4 planes × 2 eyes) that output VR image via a graphics card (Quadro FX 3800, NVIDIA Inc., USA). The participants wore passive lightweight 3D glasses coupled with circular polarization projection system of this CAVE (22).

The VR animation of the virtual ball flight was created based on the measured trajectory data collected in a setting equivalent to the PR experiment. For each speed condition of slow, medium, and fast (see “ Physical reality experiment”), the ball speed of 15 feedings was read on a radar gun (SPEEDSTER V) and then 10 data in the neighborhood of the average were chosen for making the VR stimuli. These projected balls were videotaped using two high-speed cameras (HSV-500c^3^, NAC Inc., Japan) at a sampling rate of 250 Hz and digitized manually every 10 frames (25 Hz) on motion analysis software (Frame-DIAS II, DKH Inc., Japan). The *x, y*, and *z* coordinate data of the ball flight were calculated using a direct linear transformation procedure (23).

The polygon modeling of the virtual scene was performed using 3D computer graphics software (3ds Max, Autodesk Inc., USA). The modeling parameters of virtual ball (yellow sphere of diameter 68 mm), ball machine, and experimental room were determined according to the actual scale of the PR experiment. The 3ds Max format file was converted into a stereoscopic animation format using VR visualization software (OmegaSpace, Solidray Inc., Japan; Figure 2). The binocular disparity parameter was set at 0 mm and 64 mm as the distance of eyes in the 2D and 3D display conditions, respectively. A virtual hand was modeled by an invisible sphere of diameter 100 mm and configured to move in the virtual space according to the input from a six degree-of-freedom (6-DoF) motion tracker with a maximum sampling rate of 144 Hz (Flock of Birds, Ascension Technology Inc., USA). A real-time collision detection function of OmegaSpace was enabled to assess the moment of virtual ball-hand collision: If it output “true,” the trial was judged as correct catch. The refresh rate of the VR animation and the sampling rate of collision detection were 60 Hz. A total of 20 trials (10 ball flight data × 2 random repeat) were included in the individual speed conditions, and the interval of ball feed was randomly arranged in the range of 5.00 s−5.50 s. The procedure of these VR simulations was executed by a script of OmegaSpace.

A photodetector was set in front of the right-side wall of the CAVE to detect a white rectangular image that appeared simultaneously at the moment of virtual ball launch. The 6-DoF motion tracking sensor (Flock of Birds) was embedded into a styrofoam ball of diameter 60 mm (dummy ball) that was attached onto the palm of the participants' catching hand. Data collection of surface EMG and shoulder flexion angle was conducted in the same manner with the PR experiment.

### Task and procedure

#### Physical reality experiment

The participants were seated in a chair in such a way that their shoulder of the catching arm (left: non-dominant) was located approximately 0.1 m below the target of projected ball (Figure 1). They were instructed to wait for the ball launch with the catching arm relaxed beside their body and then start the catching motion by raising the arm. The experiment of each ball speed condition (slow, medium, and fast) began with a habituation session with five trials followed by a main session of 20 trials after a few minutes of rest. The participants took an ~5-min rest period between each experiment. The order of the ball speed conditions was counter-balanced among participants.

#### Virtual reality experiment

The instructions to the participants regarding seated posture and motor task were given in a similar manner to the PR experiment. In addition, they were also asked to grasp the dummy ball on their palm at imaginary timing of ball-hand contact. In the VR experiment, two display environment conditions (VR-2D and VR-3D) were set in addition to the ball speed conditions (slow, medium, and fast). The number of trials consisted of five in habituation session and 20 in main session, which were the same as the PR experiment. The order of the ball speed conditions and the display environment conditions was counter-balanced among participants. After each display environment condition, the participants were asked to evaluate their subjective impression regarding the sense of presence about the presented virtual scene using a 101-step visual analog scale (VAS) on a laptop PC: (1) “sense of 3D effect” was feeling of depth as stereoscopic effect (weak-strong), (2) “sense of ball speed” was feeling about the speed of virtual ball as compared with that in the PR experiment (slow-fast), (3) “sense of ball dynamics” was feeling about the naturalness of virtual ball flight as expected projectile motion (unnatural-natural).

### Data processing

**Figure 3:**
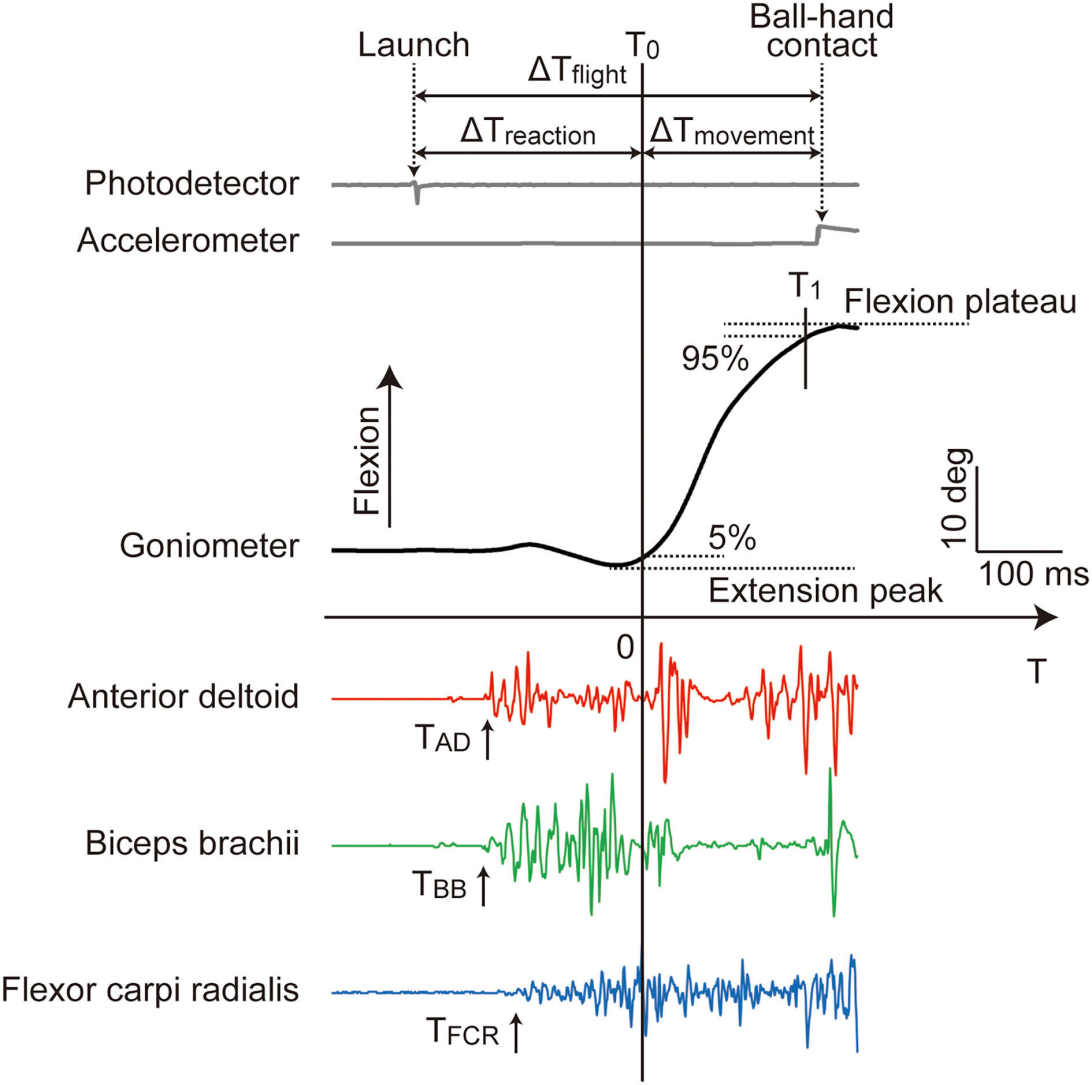
Typical profile of the analog data recorded in the PR experiment. T_0_ (T = 0) and T_1_ were determined by the 5 and 95% thresholds of the shoulder flexion total amplitude from the extension peak to the flextion plateau, respectively. All of the EMG onsets (T_AD_, T_BB_, and T_FCR_) appeared before T_0_. The ball flight time (ΔT_flight_), arm reaction time (ΔT_reaction_), and arm movement time (ΔT_movement_) were elapsed time defined as shown. The shoulder flexion velocity (ω_flexion_) was calculated as the average angular velocity over ΔT_movement_. Among these variables, ΔT_flight_ and ΔT_reaction_ were not comparable between the PR and VR experiments (see main text).

All analog data were processed off-line using MATLAB (MathWorks Inc., USA) to derive the velocity and temporal variables (Figure 3). The ball flight time (ΔT_flight_) was defined as the time elapsed from ball launch to ball-hand contact, measured using photodetector and accelerometer in the PR experiment, respectively^1^. The goniometer data of shoulder extension/flexion were filtered with a 10 Hz low-pass, second-order, zero-lag Butterworth filter. The moment of shoulder flexion initiation (T_0_: T = 0) was determined as the moment at which the shoulder angle exceeded a threshold of 5% of the amplitude from the slight extension immediately before raising arm to the flexion plateau around the ball-hand contact (Figure 3)^2^. Likewise, the moment of shoulder flexion termination (T_1_) was determined by a threshold of 95%. The arm reaction time (ΔT_reaction_) was defined as the time elapsed from ball launch to shoulder flexion initiation (= T_0_). The arm movement time (ΔT_movement_) was the time elapsed from T_0_ to T_1_. The shoulder flexion velocity (ω_flexion_) of the catching arm was obtained by calculating the average angular velocity of shoulder flexion over the arm movement time (= ΔT_movement_). These velocity and temporal variables were averaged over the 20 repeated trials to represent the individual conditions.

The EMG signals were rectified and filtered with a 50 Hz low-pass, second-order, zero-lag Butterworth filter. The onsets of AD, BB, and FCR muscles (T_AD_, T_BB_, and T_FCR_, respectively) were determined using an approximate generalized likelihood ratio algorithm (24, 25) taking the time origin at T_0_ (Figure 3). The onsets were automatically detected using a MATLAB program and, thereafter, visually inspected for each muscle in each trial and manually corrected if needed.

Statistical tests were performed using IBM SPSS Statistics 25 (IBM, USA) with the factors of environment (PR, VR-2D, and VR-3D) and ball speed (slow, medium, and fast). However, the PR condition was not included in an ANOVA for the percentage of correct catches because the requirement of task was essentially different from the VR conditions: Successful trial in PR needed physical ball grasping whereas that in VR was judged only by collision detection function of VR software. Another ANOVA for ΔT_reaction_ also eliminated the PR condition because the detection of ball launch was technically incomparable with the VR conditions (see “Apparatus and setting”). The percentages of correct catches and VAS scores were normalized by an arcsine transformation for statistical tests. In paired *t*-tests for VAS scores, Cohen's *d* was calculated as a measure of effect size. In repeated measures ANOVAs, if Mauchly's test of sphericity indicated a violation of the sphericity assumption, a Huynh–Feldt correction was applied to adjust the degrees of freedom. *Post-hoc* pairwise comparisons were made using paired *t*-tests with Bonferroni correction. Furthermore, partial eta-squared ($\eta_{\text{p}}^{2}$) was collected as a measure of effect size. The significance level was set at α = 0.05.

## Results

**Figure 4:**
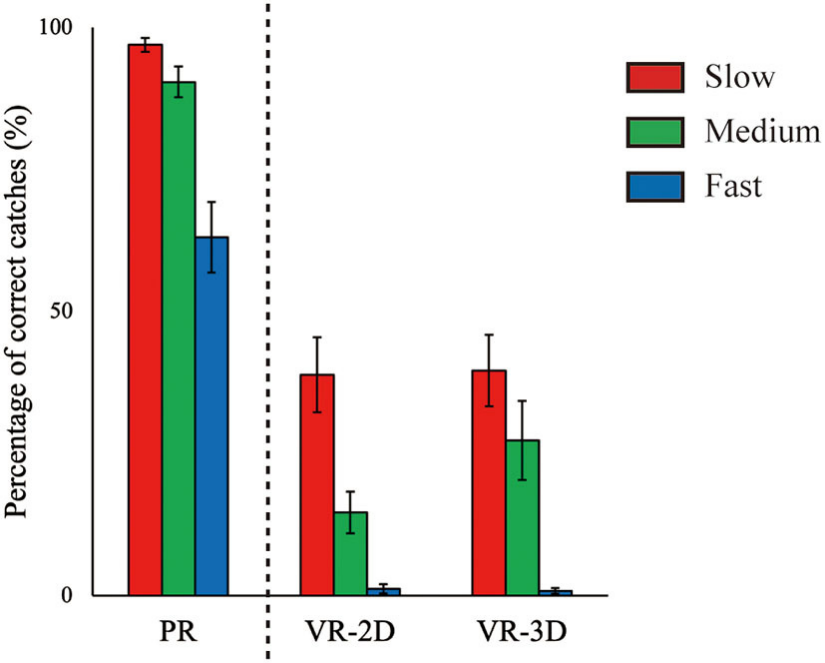
Mean (error bar: SE) of the percentage of correct catches. The PR condition, shown here for visual comparison with the VR conditions, was excluded in statistical test due to imcomparability in task requirement (see main text).

In the followings, the reports on simple effect of ball speed after observing a significant interaction are omitted to avoid redundancy as it is similar with the results in main effect of ball speed. There was no interaction and no main effect of environment in the percentage of correct catches, whereas the main effect of ball speed was significant, *F*_(2, 24)_ = 41.23, *p* < 0.001, $\eta_{\text{p}}^{2}$ = 0.775 (Figure 4). The percentage of correct catches significantly decreased with increasing ball speed.

**Figure 5:**
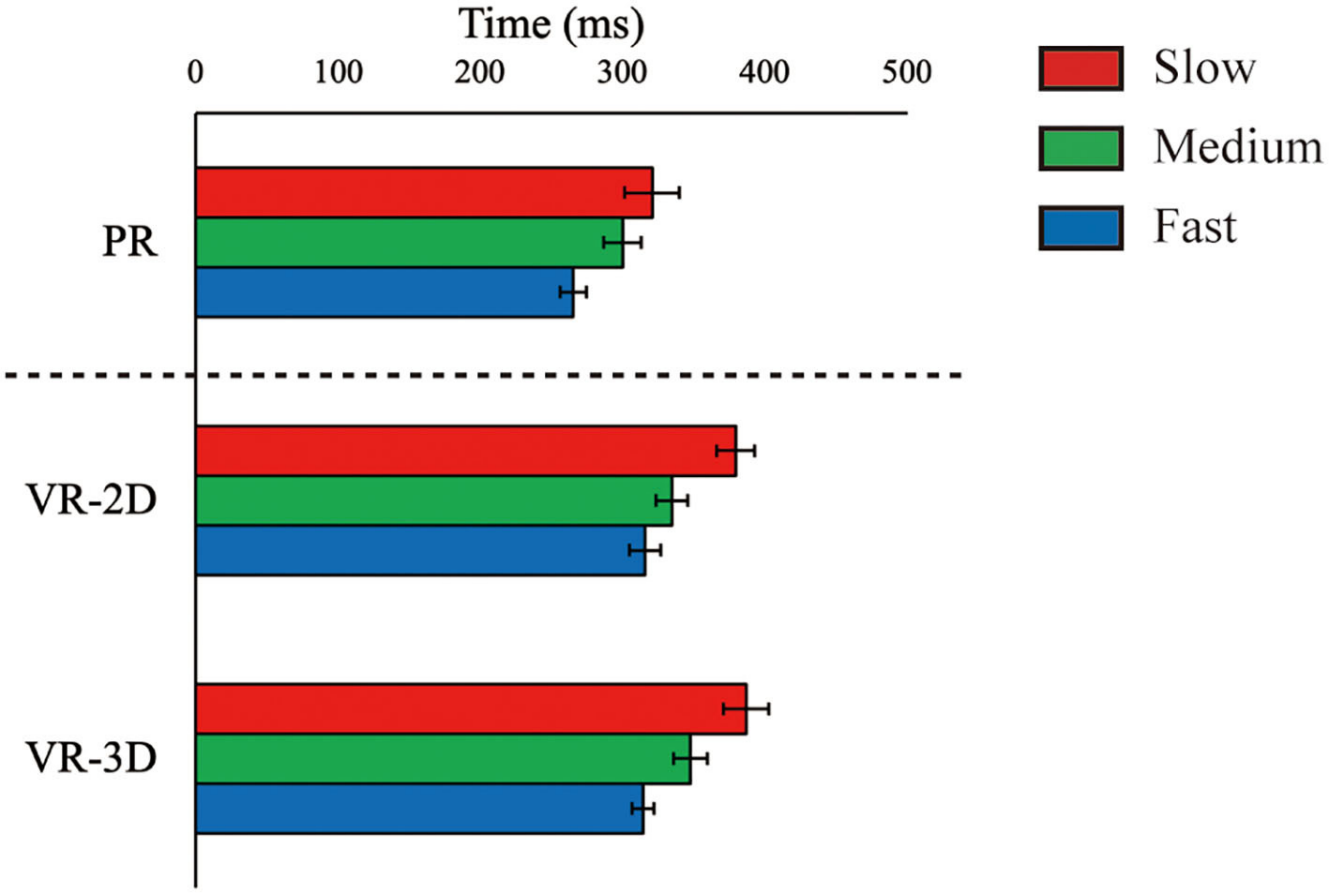
Mean (error bar: SE) of the arm reaction time (ΔT_reaction_). The PR condition, shown here for visual comparison with the VR conditions, was excluded in statistical test due to technical imcomparability in data recording (see main text).

**Figure 6:**
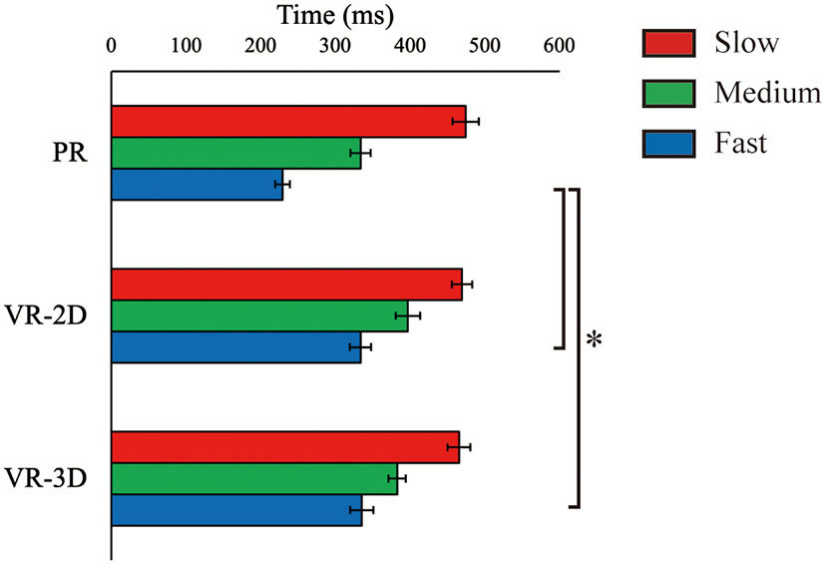
Mean (error bar: SE) of the arm movement time (ΔT_movement_). The asterisk (*) indicates the significant pairwise differences (*p* < 0.05) after a significant interaction between environment and ball speed was found.

ΔT_flight_, estimated only in the PR condition, was 883.5 ms (SE = 5.5), 682.2 (SE = 3.6), and 517.9 (SE = 2.5) for the slow, medium, and fast ball speed conditions, respectively. There was no interaction and no main effect of environment in ΔT_reaction_, whereas the main effect of ball speed was significant, *F*_(2, 24)_ = 65.61, *p* < 0.001, $\eta_{\text{p}}^{2}$ = 0.845 (Figure 5). ΔT_reaction_ was significantly shortened with increasing ball speed. There was a significant interaction in ΔT_movement_, *F*_(4, 48)_ = 10.90, *p* < 0.001, $\eta_{\text{p}}^{2}$ = 0.476 (Figure 6). Subsequently, it was demonstrated that ΔT_movement_ in the VR-2D and VR-3D conditions was significantly longer than that in the PR condition at the fast ball speed condition. In addition, there was a significant main effect of environment, *F*_(1.34, 16.06)_ = 6.08, *p* = 0.018, $\eta_{\text{p}}^{2}$ = 0.336, whereas no significant pairwise differences were detected. A significant main effect of ball speed was found, *F*_(2, 24)_ = 280.04, *p* < 0.001, $\eta_{\text{p}}^{2}$ = 0.959, where ΔT_movement_ was significantly shortened with increasing ball speed.

**Figure 7:**
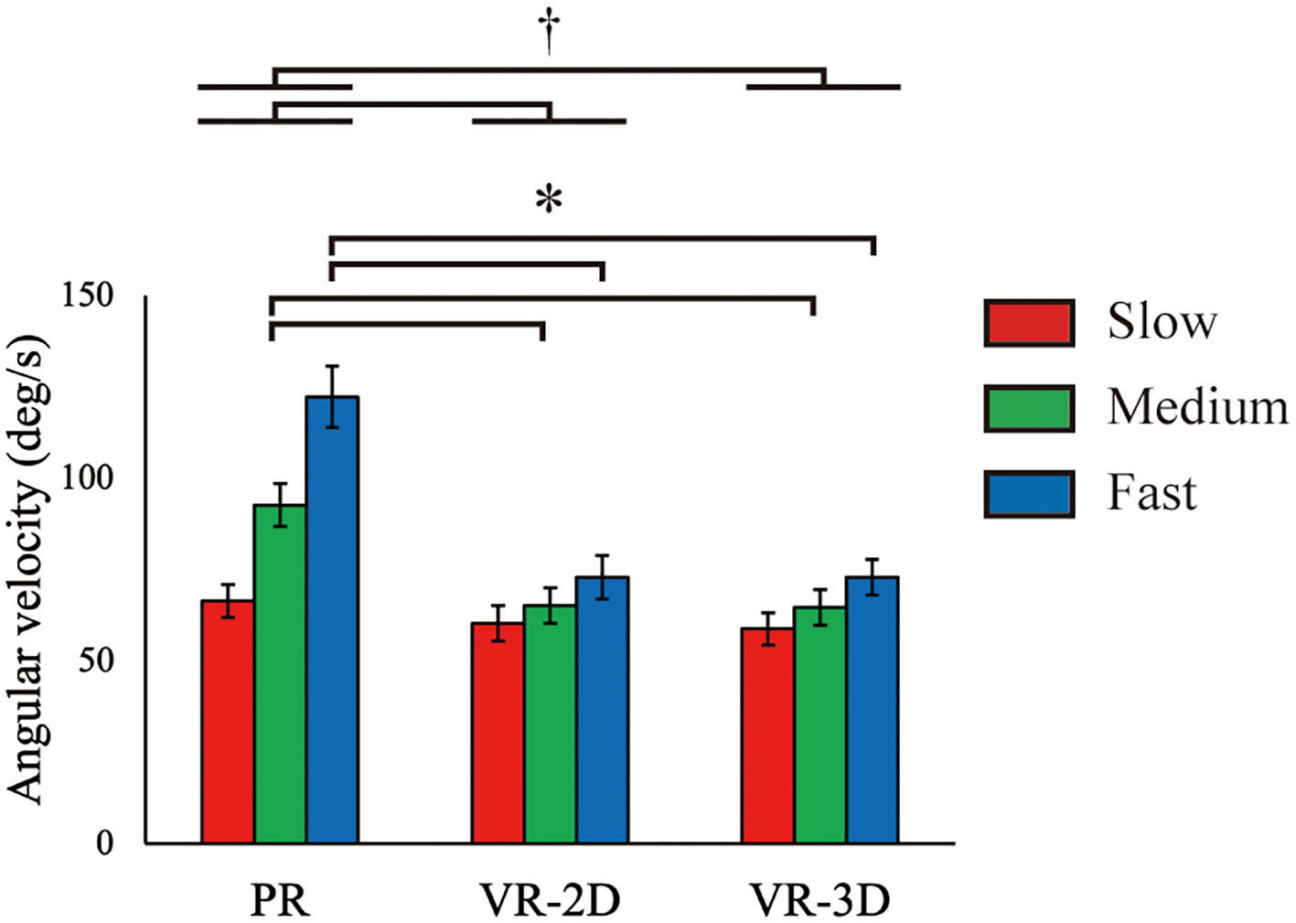
Mean (error bar: SE) of the shoulder flexion velocity (ω_flexion_). The asterisk (*) indicates the significant pairwise differences (*p* < 0.05) after a significant interaction between environment and ball speed was found. The dagger (†) indicates the significant pairwise differences (*p* < 0.05) after a significant main effect of environment was found.

There was a significant interaction in ω_flexion_, *F*_(4, 8)_ = 16.43, *p* = 0.001, $\eta_{\text{p}}^{2}$ = 0.578 (Figure 7). Subsequently, it was demonstrated that ω_flexion_ in the VR-2D and VR-3D conditions was significantly lower than that in the PR condition at the medium and fast ball speed conditions. In addition, there was a significant main effect of environment, *F*_(1.17, 14.07)_ = 18.33, *p* < 0.001, $\eta_{\text{p}}^{2}$ = 0.604; thereafter, it was revealed that ω_flexion_ in the VR-2D and VR-3D condition was significantly lower than that in the PR condition. A significant main effect of ball speed was found, *F*_(1.49, 17.82)_ = 80.45, *p* < 0.001, $\eta_{\text{p}}^{2}$ = 0.870, where ω_flexion_ significantly increased with increasing ball speed.

**Figure 8:**
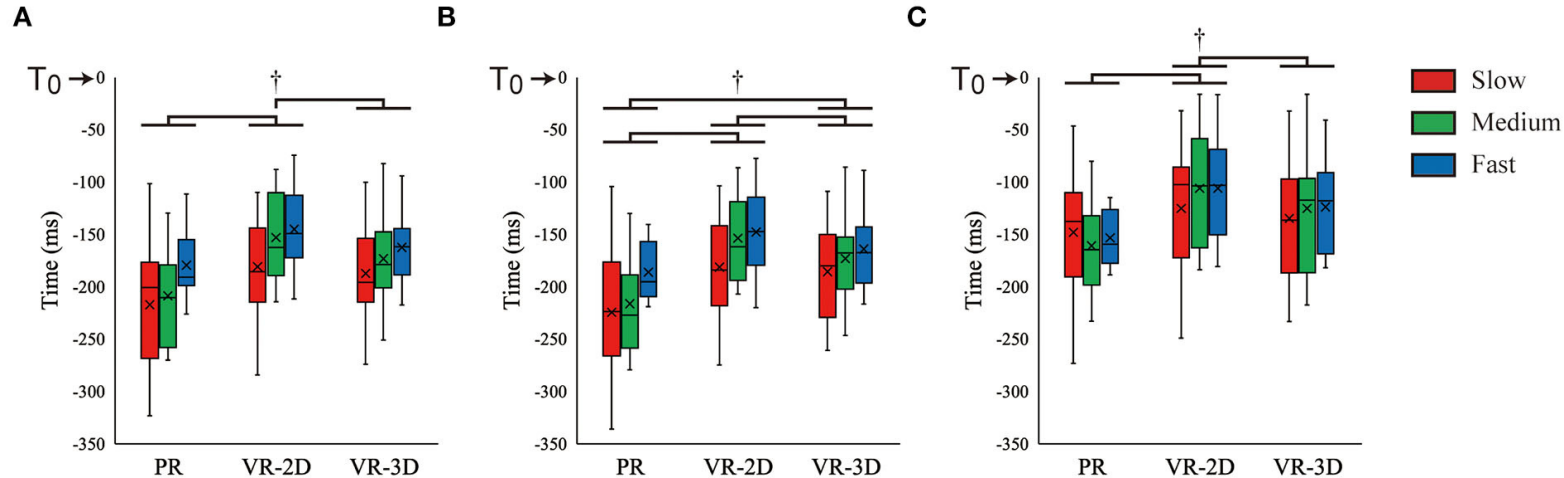
Box plot of the EMG onsets of anterior deltoid [T_AD_; **(A)**], biceps brachii [T_BB_; **(B)**], and flexor carpi radialis [T_FCR_; **(C)**] muscles. The dagger (†) indicates the significant pairwise differences (*p* < 0.05) after a significant main effect of environment was found.

There was no interaction in T_AD_. However, a significant main effect of environment was found, *F*_(2, 24)_ = 10.02, *p* = 0.001, $\eta_{\text{p}}^{2}$ = 0.455; thereafter, it was indicated that T_AD_ in the VR-2D condition occurred significantly later than that in the PR and VR-3D conditions (Figure 8A). In addition, there was a significant main effect of ball speed, *F*_(2, 24)_ = 16.62, *p* < 0.001, $\eta_{\text{p}}^{2}$ = 0.581, where T_AD_ became significantly later with increasing ball speed. T_BB_ showed no interaction but did a significant main effect of environment, *F*_(2, 24)_ = 15.20, *p* < 0.001, $\eta_{\text{p}}^{2}$ = 0.559, and a significant main effect of ball speed, *F*_(2, 24)_ = 17.32, *p* < 0.001, $\eta_{\text{p}}^{2}$ = 0.591 (Figure 8B). Subsequently, it was revealed that T_BB_ was significantly different in the order of PR (earlier) < VR-3D < VR-2D (later). Besides, T_BB_ occurred significantly later with increasing ball speed. T_FCR_ showed no interaction but did a significant main effect of environment, *F*_(2, 24)_ = 6.70, *p* = 0.005, $\eta_{\text{p}}^{2}$ = 0.358 (Figure 8C). It was demonstrated that T_FCR_ in the VR-2D condition was significantly later than that in the PR and VR-3D conditions.

The sense of 3D effect was significantly stronger in the VR-3D condition than in the VR-2D condition, *t*_(12)_ = 1.79, *p* = 0.049, *d* = 0.610. There were no significant differences in the sense of ball speed, *t*_(12)_ = 1.62, *p* = 0.066, *d* = 0.424, and in the sense of ball dynamics, *t*_(12)_ = 0.24, *p* = 0.406, *d* = 0.072.

## Discussion

This study was designed to determine the discrepancies among the display environments (PR, VR-2D, and VR-3D) in the motor responses of one-handed ball catching examined with three ball speeds (slow, medium, and fast). The primary hypothesis of this study was verified by the findings that the EMG response and resulted shoulder motion in the two VR conditions were different from those in the PR condition. More specifically, the VR conditions brought the EMG onsets closer to T_0_, slowed the shoulder flexion velocity, and elongated the arm movement time as compared with the PR condition. However, this elongation in movement time was the opposite result to the initial expectation of the current study. The secondary hypothesis was partially supported by the findings that the differences between the VR-2D and VR-3D conditions were observed in EMG response. Specifically, the EMG onsets in the VR-2D condition occurred later than those in the VR-3D condition. The supplementary hypothesis also confirmed that the effects of ball speed in one-handed catching task were consistent between PR and VR. To be specific, the percentage of correct catches decreased, and the arm reaction and movement times were shortened with increasing ball speed.

In a previous study that examined the performance of judging and intercepting fly balls in CAVE, it was concluded that the catchers' moving path in the VR setting resembles the way they would do so under natural conditions (13). The current study, in contrast, showed that the motor responses of the catching arm were substantially different between natural environment of PR and simulated environment in CAVE. In particular, the arm movement time was longer in the CAVE environment than in the PR environment. This was an opposite outcome to another previous study, which pointed out that the elapsed time of hand movement was shortened in a ball-catching task performed in CAVE than in an equivalent PR setting (14). One possible reason for these contrary results was the difference of ball flight pattern presented: the previous study used a pendular movement in which the ball was suspended from the ceiling, whereas the current study did a parabolic movement in which the ball was projected from a ball machine. Another reason was the difference in the requirement of motor task: the previous study restricted the hand movement only in the lateral direction along a horizontal bar, whereas the participants of the current study were instructed to raise their arm from a lowered position.

The onsets of all the muscles examined in the current study occurred significantly later in the VR-2D condition than in the PR condition. Likewise, a significantly later onset was observed at BB muscle in the VR-3D condition as compared with in the PR condition. In particular, the duration from the firing of AD muscle (T_AD_) to the initiation of shoulder flexion (T_0_) is referred to as motor time that is the latter fraction of reaction time after the former fraction of premotor time, for raising the catching arm (20). The motor time is essentially constant if the experimental conditions are identical in terms of properties of the body segment inertia and internal/external force (20, 26). The shortened motor time for shoulder flexion in the VR-2D condition appeared to be attributed in part to the difference in internal force from the PR condition. More specifically, this denotes that the force patterns generated by the agonist and antagonist muscles may be altered due to the environmental effect of VR.

Furthermore, differences between the VR-2D and VR-3D displays were found in the EMG onsets and in the VAS score on the sense of 3D effect. In addition, there was marginal difference in the percentage of correct catches between these two VR conditions (p = 0.058) and in the VAS score on sense of ball speed (*p* = 0.066). Although further investigation is needed whether the 3D display induces a higher chance of successful catch than the 2D display, at least, it is suggested that the muscle activation pattern of the catching arm can be differentiated by these display settings. The findings of the current study partially support the notion that the binocular disparity information of approaching ball contributes to the control of interceptive action (9, 10). These previous studies clearly demonstrated the difference in catching performance by comparing the groups of normal and weak stereopsis. However, since there have been few studies on the display effect of 2D or 3D, the findings of the current study will provide new insights into the effect of monocular and binocular information on the control of interceptive actions.

A potential psychophysical factor that alters the motor responses of the catching arm is size misperception of virtual space introduced by VR, known as perceptual distortion (27). For example, a number of studies using HMD reported that the perceived virtual space was compressed (28), although there were some exceptions that induced expansion (29). Such perceptual distortion is considered to be one of the primary factors that causes an underestimation in the time to contact of approaching object (30, 31). On the contrary, it has been shown that observers of an approaching fly ball presented in HMD are able to predict the position of the ball and time to contact based on limited visual information of its parabolic flight (32). This prediction may enable VR users to simulate the catching motion in a similar manner as they performed in PR environment. However, the results of the current study implied that there were substantial differences between PR and VR for the perception–action coupling in ball catching.

The current study demonstrated that a simulated virtual environment did induce a different neuromuscular control from a physically real situation while catching a ball. In particular, the onsets of the catching arm muscles were different between 2D and 3D virtual displays as well as between the real and virtual situations. A previous study concluded that there was little difference in the success rate of one-handed catching between dominant and non-dominant hand; thus presumably, the findings of this study can be applied to either hand (33). The natural task used in the current experiment might be responsible for the large inter- and intra-personal variability (9) as compared with highly controlled setup such as a pendular projectile (14). Nevertheless, the current study would provide practical insights into neuromuscular control of catching in the context of ecologically valid real-life situation or parabolic flight of ball. Further exploration is expected to clarify how the perception–action coupling between the visual information sources presented in VR and the motor responses found in end effector are altered not only in terms of kinematic patterns but also in terms of muscle activities.

## Data availability statement

The original contributions presented in the study are included in the article/supplementary material, further inquiries can be directed to the corresponding author.

## Ethics statement

The studies involving human participants were reviewed and approved by Jobu University and Kanagawa Institute of Technology. The patients/participants provided their written informed consent to participate in this study.

## Author contributions

HI and KF contributed to conception and design of the study. KF and TO created the visual stimuli. HI and TO organized the experiment. HI performed the data analysis and wrote the first draft of the manuscript. All authors contributed to manuscript revision, read, and approved the submitted version.

## Funding

HI and KF were funded by the Japan Society for the Promotion of Science (JP19H03982 and JP18K10890, respectively).

## Conflict of interest

The authors declare that the research was conducted in the absence of any commercial or financial relationships that could be construed as a potential conflict of interest.

## Publisher's note

All claims expressed in this article are solely those of the authors and do not necessarily represent those of their affiliated organizations, or those of the publisher, the editors and the reviewers. Any product that may be evaluated in this article, or claim that may be made by its manufacturer, is not guaranteed or endorsed by the publisher.

## Footnotes

^1^The ball flight time was not determined in the VR experiment due to the lack of physical contact between ball and hand.

^2^The time of ball launch was not set as the time origin (T_0_) because it was technically incomparable between the PR and VR experiments due to the difference of photodetector setting.
